# Towards insulin independence in type 1 diabetes: Prospects for prevention and cure

**DOI:** 10.1371/journal.pmed.1004813

**Published:** 2025-11-25

**Authors:** Guy I. Sydney, Ana Luisa Perdigoto, Kevan C. Herold

**Affiliations:** 1 Section of Endocrinology, Department of Internal Medicine, Yale School of Medicine, New Haven, Connecticut, United States of America; 2 Department of Immunobiology, Yale School of Medicine, New Haven, Connecticut, United States of America; 3 Department of Internal Medicine, VA Connecticut Healthcare System, West Haven, Connecticut, United States of America

## Abstract

This Perspective highlights exciting developments in preventative treatments and stem cell-based therapies for type 1 diabetes, discussing the prospects and hurdles of arresting or reversing the disease and achieving insulin independence for type 1 diabetics.

## Teaser

The discovery of insulin transformed type 1 diabetes from an acutely lethal illness to a chronic disease that is managed with insulin dependence. Now, exciting developments in preventive treatments and stem cell-based therapies bring the prospects of arresting the disease and achieving insulin independence for type 1 diabetics closer to reality.

## Introduction

Type 1 diabetes (T1D) is an autoimmune disease characterized by the progressive destruction of insulin-producing pancreatic beta cells, resulting in severe metabolic consequences and a lifelong dependence on exogenous insulin administration. The incidence of T1D continues to rise, with an estimated 9.5 million people living with the condition globally in 2025 [1]. Since the discovery of insulin in 1921 and for the next century, the sole therapy to manage T1D had been its replacement by exogenous administration. Over time, new formulations enabled better pharmacokinetics, and advances in technology led to semi-automated insulin administration systems. These developments have enhanced metabolic management, leading to improved prognosis and reduced risks of secondary end-organ complications.

However, exogenous insulin replacement cannot fully replicate physiologic dynamics. Non-physiologic route of administration, the continuous risk of hypoglycemia, accessibility to insulin, the cumbersome nature of the technologies, and the overall cost of disease management has driven the search for alternative therapies. In recent years, there have been exciting breakthroughs in efforts to prevent/ delay the progression of T1D, as well as the development of stem cell-based and gene editing therapies aimed at restoring beta cell function to eliminate insulin dependence. These achievements herald the beginning of a new era for T1D therapeutics, but not without their own hurdles.

## Preventing T1D before clinical presentation

**Fig 1:**
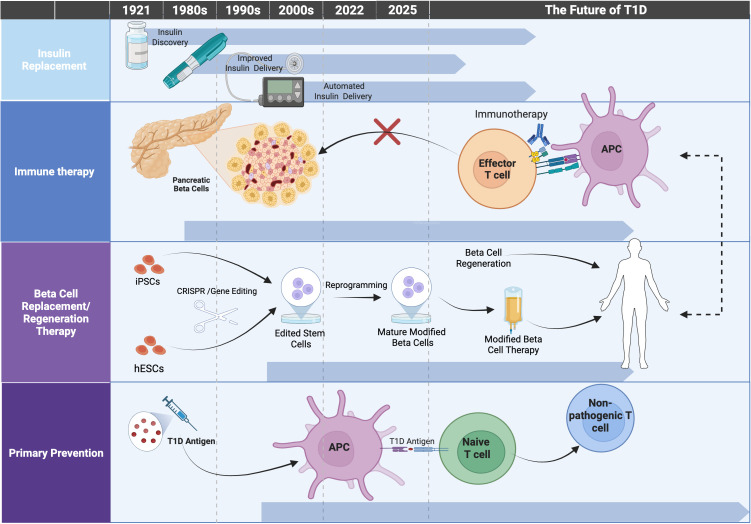
Timeline of therapeutic strategies in type 1 diabetes illustrating past, current, and emerging interventions. Historically, the clinical strategy has been metabolic management focused exclusively on exogenous insulin replacement to correct its absence after destruction of beta cells. Advances in the field have led to optimized glycemic control through advanced insulin formulations, continuous glucose monitoring, and automated delivery systems. Beginning in the 1980s, immunomodulatory therapies began testing, aimed at preventing or slowing the loss of beta cell function after clinical diagnosis. With the recognition that the pathogenesis occurs over years and that prediction of disease is possible, the delay in diagnosis in those at risk led to its approval in 2022. Emerging strategies include beta cell replacement, with genetic modifications of the transplanted cells, potentially in combination with a brief immune treatment, to avoid the need for chronic immune suppression, or even induction of beta cell regeneration. The long-term goal, which would require expansion of screening to the general population and the enhancement of rates and duration of responses, is to arrest disease before its presentation, as exemplified by teplizumab. Figure created in Biorender. Sydney, G. (2025) http://BioRender.com/ywgcezg.

T1D can be identified before its clinical presentation. It is first detected by autoantibodies in the serum, and its progression towards insulin dependence, through metabolic dysregulation measured by response to oral glucose challenge [2]. Clinical trials have focused on secondary prevention after signs of autoimmunity or after the appearance of overt hyperglycemia (Stage 3 T1D), and several agents have shown transient efficacy in modifying the disease course (Fig 1). Examples include agents that deplete B cells (e.g., rituximab) or T cells (e.g., alefacept or ATG), block inflammatory pathways (e.g., Baricitinib), or modulate the cytokines that are produced by immune cells (e.g., anti-IL-21 or TNFα) [3].

More recently, in a paradigm shift towards disease prevention, teplizumab (in 2022) became the first immunotherapy approved by the U.S. Food and Drug Administration (FDA) to delay the onset of clinical T1D in those at risk [4]. Teplizumab works by attenuating autoreactive T cells and thus preserving insulin-secreting beta cells, showing efficacy in delaying the diagnosis of T1D by 2–3 years on average, while in some by nearly a decade. However, individuals in whom autoimmunity leading to T1D has been initiated are completely asymptomatic, and can only be identified by immunologic and metabolic testing. Thus, while earlier intervention is desirable, identifying at-risk individuals is challenging and will require broad elective screening efforts. An ongoing study is evaluating clinical outcomes for treatment at the time of clinical presentation [5]. Teplizumab has been awarded a national priority voucher meant to accelerate review of the drug.

## Restoring lost beta cell mass

Loss of beta cell function in T1D is permanent, and therapeutic efforts to restore their function have shown little success to date. Moreover, even if detected prior to significant beta cell loss, not all treated patients will respond to teplizumab or other immunotherapies. Therefore, for those who have already lost beta cell mass or are unresponsive to immunotherapy, beta cell regeneration or replacement may be the most promising path towards disease reversal. The potential benefits are significant: Due to exquisite functional precision, minimal endogenous beta cell capacity can reduce severe hypoglycemia associated with exogenous insulin therapy even if insulin independence is not achieved. Therefore, for patients suffering from recurrent severe hypoglycemia, beta cell replacement may be the preferred option.

In 2000, successful transplantation of human pancreatic islets into seven T1D patients resulted in acquisition of insulin independence for all recipients [6]. Yet, original excitement was tempered due to the limited availability of cadaveric islets suitable for transplantation, diminishing long term graft survival with recurrence of insulin dependency, and most importantly, the need for continuous immunosuppression [7]. Nonetheless, after combating the technical hurdles of product variability and regulatory requirements and endpoints, Donislecel, (lntidra) became the first FDA approved cellular therapy for T1D (in 2023) for the indication of recurrent severe hypoglycemia [8].

However, to address the limited availability of cadaver islets, researchers needed to turn to another source of beta cell replacement: Stem cells (Fig 1). Advances in stem cell technologies have enabled the development of therapies in which human embryonic (ESC) or induced pluripotent (iPSC) stem cells can be differentiated in vitro into insulin-producing beta cells. These can then be transplanted into T1D patients to restore beta cell mass and function. This approach is being pioneered by trials such as VX-880 (Zimislecel), the first ESC-derived islet therapy to reach phase 3 clinical trials. In a phase 1/2 trial that enrolled patients with T1D and severe hypoglycemia, severe hypoglycemia was eliminated in all 12 who reached 1 year of follow-up, with 10 achieving insulin independence [9]. Of note, by 180 days after the transplant the glucose response to a mixed meal challenge was normal which cannot be achieved with exogenous insulin administered even by hybrid pumps. This extraordinary achievement of successful transplantation of stem cell derived beta cells is of importance, as these provide a potentially limitless source of replacement and could eliminate the need for insulin administration and concomitantly severe hypoglycemic events. However, as with cadaver islets, a major limitation is the need for life-long immunosuppression to prevent rejection.

This limitation might be overcome through genetic engineering of the cells. A recent report described the successful transplant of cadaver-derived islets in a single individual, in which the transplanted cells were genetically modified (by CRISPR/Cas12b editing) to prevent elicitation of an immune response, rather than suppressing the immune cells themselves [10]. In non-human primates, these “hypo-immune” islets achieved insulin independence [11]. In comparison, the patient received a sub-therapeutic dose of islets and hence did not achieve insulin independence. However, C-peptide (marking endogenous insulin production and which was undetectable before the transplant) was detectable for at least 12 weeks afterwards, indicating graft survival and function. Importantly, neither adaptive nor innate immune responses to the graft were detected in the recipient, despite the islets being transplanted without immune suppression.

Another method to potentially eliminate the need for immunosuppression is to utilize the patient’s own cells. In 2024, Wang and colleagues reported the clinically successful transplant and insulin independence of iPSC derived islets from a patient with T1D back into the same patient (autologous transplantation) [12]. The recipient had been on immune suppression to prevent a liver graft rejection after a transplant for unrelated reasons, but using autologous cells overcomes the barrier of allo-rejection.

## Hopes and hurdles for preventive and curative treatments reaching the clinic

Longer term safety and efficacy data will be needed for all beta cell replacement approaches. While autologous cell-based therapy strategies may prove effective and avoid allo-rejection, they risk reactivating the autoimmune process that destroyed the cells to begin with. A clear next step is to combine advancements in stem cell derived therapy with those in genetic engineering, allowing the restoration of metabolic function alongside evasion of allo or autoimmunity. Combining therapies such as teplizumab with cell replacement may also achieve this goal. This may enable much broader implementation of cell-based therapy as it would not be constrained by donor availability, the need for lifelong immunosuppression. The absence of personalization requirements could facilitate standardized manufacturing pipelines, allowing for more rapid production and reduced cost. Several questions regarding the optimal agent, timing, need for repeated drug administrations or cell transplantation, will require evaluation through clinical studies. Finally, streamlining will be needed to keep the costs of cell therapies and biologics manageable and the therapies available at the same time that the capacity for production is increased. Clearly, the costs will be significant but the impact for children, their families, and adults with T1D is invaluable.

The longer reaching goal is complete prevention (Fig 1), before clinical diagnosis, so that beta cell replacement is not needed. Prevention is a novel concept for autoimmune diseases, that will require time to allow refinement of technologies for diagnosis, biomarkers of dise/ase activity, and acceptance. Teplizumab, the first drug to achieve a delay in T1D diagnosis, sets us down an exciting path towards prevention. As the safety, efficacy, and practicalities of autoimmune therapies improve, prevention may become a reasonable goal.

## Abbreviations

FDA: Food and Drug Administration
T1D: type 1 diabetes
